# Ozone Therapy for Complex Regional Pain Syndrome: Review and Case Report

**DOI:** 10.1007/s11916-019-0776-y

**Published:** 2019-05-06

**Authors:** Robert Jay Rowen, Howard Robins

**Affiliations:** 1 Santa Rosa, USA; 2 New York, USA

**Keywords:** Reflex sympathetic dystrophy, Complex regional pain syndrome, Ozone therapy, Pain management

## Abstract

**Purpose of Review:**

The world faces a crisis in pain management. CRPS is a multifaceted painful disorder, which is difficult to treat and resolve. Ozone therapy has unique mechanisms of actions that may directly address the emerging discoveries of factors related to pathogenesis of the disorder and other pain conditions. These include oxygenation, immune modulation, anti-infective properties, and anti-inflammatory properties. This review is to present ozone therapy as a novel approach for pain treatment, including CRPS.

**Recent Findings:**

Ozone therapy has been found, in basic science studies, to ameliorate many of the mechanisms promoting chronic pain and inflammation, including hypoxia, inflammatory mediators, and infection. Direct intravenous oxygen/ozone gas was administered nearly daily to an 11-year-old girl diagnosed with reflex sympathetic dystrophy and extremely frequent pseudoseizures. She rapidly improved. After 120 sessions, all symptoms had disappeared.

**Summary:**

Ozone’s novel biochemical properties may make it a unique, safe, relatively inexpensive, and effective modality for the treatment of pain. In this particular case, it resolved the chronic condition when opiates were ineffective for even pain relief. Ozone therapy should be considered for institutional study despite its lack of financial reward (lack of patentability).

**Perspective:**

This manuscript presents ozone therapy as a novel, safe, and inexpensive approach for RSD/CRPS, and an alternative to drugs. It is practiced worldwide and has abundant literature on its biochemical mechanisms, effectiveness for pain (and other conditions), and overall healing usefulness, yet little is known conventionally as it is not patentable.

## Introduction

America has a crisis in pain management with opioid abuse and deaths rapidly rising. Healing approaches rather than chemical masking of pain are urgently needed.

Reflex sympathetic dystrophy, now known as Complex Regional Pain Syndrome, has been summarized as a condition consisting of burning pain, swelling, color and temperature changes, hyperesthesia, and hyperhidrosis in an affected extremity. Its path lies outside common dermatomes. The cause is generally a trauma, accidental physical or surgical, which deranges the autonomic nervous system, causing distant effects. It can spread to other extremities, absent additional trauma [1].

It is a difficult condition to treat. Goh et al. report that traditional options include physical and occupational therapy, psychological therapy, surgical ablation, drugs (including corticosteroids), sympathetic blockade, bisphosphonates, DMSO, antioxidant therapy, sympathectomy, and even limb amputation. “Emerging treatment” includes immunomodulation (due to the presence of inflammatory cytokines), botulinum toxin, and hyperbaric oxygen (though the report on this approach was using the modality shortly after the clinical precipitating injury). NSAID drugs are of little value [2]. Hyperbaric oxygen may work through a prolonged NO-dependent release of opioid peptideantinociceptive effect [3]. Hyperbaric oxygen therapy induced rapid pain relief. VAS scores markedly improved by the end of the first day. It also was effective at reducing edema while improving range of motion [4]. This fits with the concept of the condition being sympathetic nerve vasoconstriction mediated. Nitric oxide (NO) is a vasodilator, and hyperbaric oxygen will assure oxygen delivery to hypoxic tissues, even tissues with reduced vascular circulation.

Medical ozone is a mixture of oxygen/ozone gas, typically 1–5% O_3_ and 95–99% O_2_, and is the “ozone” referred to in this report. Ozone in nature is the strongest naturally occurring oxidant. Surprising findings at Scripps has determined that human neutrophils can generate ozone in the body by a catalytic reaction of immune cell endogenously created singlet oxygen and antibodies [5]. Medical “ozone” is generated by passing medical oxygen over a variable corona arc discharge, creating ozone in a desired concentration. It has been in continuous medical use for over 100 years, used by German soldiers in the trenches to disinfect wounds in WWI. Recent research both in vitro and in vivo demonstrate ozone to have powerful immune modulating and healing effects. In addition, it virtually instantly destroys bacteria and viruses some 150× faster than bleach [6, 7], another oxidant. It is extensively used worldwide via local injections to joints [8] and discs [9] for the resolution of arthritis and pain. It is extremely safe. A 1982 German report cites a complication rate of just 0.7 per 100,000 treatments, with virtually all complications secondary to improper administration [10]. Since then, there have been no credible reports of ozone therapy harm to patients.

## Ozone Biochemistry

Research teams led by Bocci in Italy, and Menendez in Cuba, have independently found the following published effects summarized in their books [11, 12]. The therapy modulates the immune system via balancing inflammatory and anti-inflammatory cytokines, increases production of RBC 2, 3 DGP (stimulating more oxygen delivery), improves red cell rheology, increases anti-oxidant enzymes, and increases glutathione production. These effects are accomplished by ozone’s induction of reactive oxygen species and lipid oxygen products known as ozonides. These last much longer than ozone, which reacts with blood products virtually instantly. There is no chemical residue left for the body to metabolize, which drugs leave behind, as ozone is oxygen.

Menendez’s group found ozone therapy equivalent to dexamethasone in attenuating TNF-α [13]. Ozone therapy may be superior to drug therapy and even chemical lumbar sympathectomy and epidural injections for limb vascular pain and ulcers [14]. Ozone has been found superior to hyperbaric oxygen therapy in improving blood rheology. While HBO therapy had no effect on blood viscosity, ozone therapy did significantly reduce viscosity [15], an important factor in flow.

Ozone has many methods of administration. Common methods of blood administration include direct intravenous gas (DIV), major autohemotherapy (MAH), and hyperbaric ozone therapy (HBO_3_). The latter two involve removing an aliquot of blood, adding ozone, and reinfusing to the patient. Hyperbaric ozone therapy adds the ozone to the blood under pressure, while MAH returns ozonated blood under gravity.

DIV ozone is administered commonly throughout the world as it is exceptionally inexpensive, requires little medical equipment, and generates minimal waste. The method has been refined by Dr. Robins and is performed as follows. An aliquot of oxygen/ozone gas of desired concentration is withdrawn into a syringe from an ozone generating machine. It is slowly administered intravenously over several minutes. Generally, the concentration is 55 μg/cc. The adult volume usually begins at 20 cc and is gradually increased up to 100 cc as tolerated by the patient (discussed below).

Intravenous oxygen gas administration is not new. European practitioners have used it for decades and reported beneficial effects. Regelsberger observed a general improvement in oxygen availability, and eosinophilia, which can be valued as an increase in undetermined cellular immunological resistance. “Furthermore, rheological qualities of the blood as well as diuresis are improved, the release of oxygen into the tissue is increased, and the blood pH is normalized” [16]. Intravenous oxygen gas in human volunteers induces eosinophil generated 15-LOX-1, a powerful anti-inflammatory enzyme, believed to be a key factor in the inflammatory-modulating effects of IV oxygen gas [17]. Intravenous oxygen gas also increases the all-important prostacyclin/thromboxane ratio [18].

The foregoing suggests that ozone therapy might offer hope for RSD/CRPS.

## Case Report

The patient was an 11-year-old female who presented to the emergency department and was admitted to the hospital in October 2016 with a 1-month history of extreme right lower extremity burning pain and seizure-like activity. MRI of back and leg were negative save for inflammation noted in the ligaments around the right knee. EEG was performed because of very frequent episodic seizure-like activity in conjunction with the pain. It was negative, so diagnosis of pseudoseizures was made. She had been treated with several drugs for the pain, including Toradol, Motrin, Tylenol, Oxycontin, and even IV morphine in the emergency department. Psychogenic causes were evaluated. Her children’s hospital records suggested reflex sympathetic dystrophy, considered because of alterations in color and temperature of her right leg, and evidence of inflammation in peri-knee ligaments.

Videos taken of the patient show her writhing in severe pain and with apparent seizure activity. After 3 months of these unrelenting symptoms (pain and pseudoseizures), she presented to the office of Dr. Robins. Informed consent was obtained. Robins provided ozone therapy by the method of direct intravenous gas (DIV). She received five treatments per week, for 26 weeks for a total of 120 treatments. She began therapy at 5 cc gas at a concentration of 55 μg/cc and was gradually increased to 30 cc at 55 μg/cc.

After 10 sessions, her pain improved and pseudoseizures diminished from 25 to 30 per day to 12–15 per day lasting ½ the previously average time of 2–3 min. After 3 months, pseudoseizures became rare and by the 4th month (120 sessions) had stopped completely, along with clearance of all pain. She returned to school symptom-free 1 year after it had begun and has stayed symptom-free since.

## Discussion

Considering the current thought on CRPS mechanisms, ozone therapy may be an ideal approach. The therapy addresses many of the proposed abnormalities in the syndrome (inflammation, nitric oxide deficiency, reduced circulation). The newer conventional medicine treatments include immune modulation and hyperbaric oxygen. Bocci and Menendez have demonstrated ozone’s ability to modulate the immune system. Bocci called ozone “the ideal cytokine inducer” and believes that ozone therapy creates “super gifted red cells” (personal communication).

Hyperbaric oxygen is theorized to improve CRPS through NO. Bocci’s group found endothelial cell NO induction by ozone therapy [19]. The overactivity of the sympathetic nervous system, from whatever insult, likely compromises circulation and oxygen delivery to affected tissues. NO vasodilates. Other ozone metabolic effects clearly enhance oxygen delivery and metabolism. In fact, it has been found to increase the Pa-Pv O_2_ difference [12]. This could only be accomplished through increased oxygen delivery and mitochondrial uptake and combustion to energy. The combined biochemical effects of ozone therapy suggest the modality may carry a more powerful effect on CRPS than hyperbaric oxygen.

Pseudoseizures are episodes that resemble epileptic seizures and are often misdiagnosed as epilepsy. However, they are considered of psychological origin, such as stress (https://emedicine.medscape.com/article/1184694-overview), which could include experiencing pain.

The patient steadily improved, over time, to full recovery. We believe it is unlikely to be placebo effect. She had been hospitalized, examined thoroughly with studies and psychologically, given drugs of all kinds, including opiates, with no improvement and limited, temporary relief for the pain. The weakness of this report, of course, is a series of one. The strength is the possibility of a very inexpensive and safe method of treatment for a very difficult condition to treat. Additionally, ozone therapy was the single therapy employed after her hospitalization leading to recovery.

Author Rowen, together with his trainees in ozone therapy, has treated many painful conditions with ozone therapy, including apparent resolution of CRPS by injecting the primary site of injury with ozone gas. Most ozone treatments for pain do involve the gas injected directly into diseased/injured tissues, such as joints. However, we have consistently observed significant reductions in body pain, both generalized and local, using systemic blood ozonation by the HBO_3_ technique. As stated, DIV ozone is simpler, easier to do, less expensive, and generates minimal medical waste. DIV has the added advantage over hyperbaric oxygen of requiring much less office space, far cheaper equipment, and negligible treatment time.

## Issues Regarding DIV Ozone

DIV ozone is controversial among ozone practitioners. Its downside is vein irritation (veins lack catalase protection), and it can induce several minutes of cough and chest tightness if administered in excess and too fast. However, Dr. Robins has personally performed or supervised over 250,000 treatments absent of any significant or lasting untoward effects except local venous issues. It is for the vein issue that HBO_3_ therapy is used by seasoned ozone therapists when such therapy is available and there is good vein access. (HBO_3_ therapy requires a larger needle/catheter, but since the ozone is added to the blood outside the body, where it instantly reacts with the blood, it has no oxidative endothelial impact on the vein). We used DIV ozone in the 2014 West Africa Ebola epidemic, where the method remitted 5 of 5 cases of acute Ebola in an epidemic killing 60% of its victims [20]. Its ease of availability and extraordinary low cost (cost of butterfly needle and syringe only, after ozone generator and gas supply is secured) have made the DIV method likely the most used in the world (personal knowledge), especially outside Europe and North America. However, we both prefer the HBO_3_ method where there is good venous access, based on the latter’s overall clinical results, and lack of irritation to the vein.

The thought of DIV gas is anathema to many physicians, fearing “air” embolism. Air is 80% nitrogen. Medical ozone is 100% oxygen, a metabolizable gas which will rather rapidly be up taken by O_2_ thirsty red cells. Its long use in Europe demonstrates its safety. The vein irritation in DIV ozone is not the oxygen, but the oxidative effects of the ozone on catalase-deficient vein endothelial cells [21]. Our experience demonstrates higher concentrations of ozone in the procedure are more vein irritating. Both the volume of gas and concentration can be adjusted depending on patient response and signs of excess (such as vein irritation and/or chest tightness and coughing).

Unfortunately, it is costly for any therapy to be “approved” by the FDA, to the astronomical cost of literally billions of USD (https://www.policymed.com/2014/12/a-tough-road-cost-to-develop-one-new-drug-is-26-billion-approval-rate-for-drugs-entering-clinical-de.html). Ozone cannot be patented for profit. There is no incentive to invest in, and find successful therapies, including ozone, when dealing with such a financial abyss. This “non-approved” status is the reason ozone therapy is largely ignored by mainstream medicine. And, ozone therapy suffers from the “tomato effect,” described in JAMA where a highly efficacious therapy is ignored or rejected because it just does not make sense in light of prevailing medical thought [22].

We consider the origin of pain to be a combination of injury, degeneration, or non-infectious or subclinical infectious inflammation, and reduced oxygen availability, all of which will impair energy production for tissue repair and healing. The source of pain can be local or distant. Ozone therapy addresses both inflammation and oxygen availability, and infection as well. Together, the authors have 60 years of ozone therapy experience. Save a few ultra-short prescriptions for acute injury and/or compassionate relief of cancer pain, neither of us has written a new prescription for opioids in 20 years. We credit ozone therapy, which we see can treat and heal many causes of pain. No one will dispute oxygen as being the most important need in healing.

## Conclusion

Ozone therapy has a long history of use in medicine, from circulatory improvement to treatment of infection and pain, but is very new and novel to conventional medicine. Its researched biochemical mechanisms suggest it to be a worthy consideration for CRPS. This case in a young girl, who had been unsuccessfully treated with opiates for her pain, demonstrates its possibilities. Non-drug treatments for pain are urgently needed in this era of opioid crisis. Ozone therapy deserves active study in pain centers for treatment of CRPS and other conditions of pain.
